# Native myeloperoxidase is required to make the experimental vasculitis model

**DOI:** 10.1186/s13075-019-2084-7

**Published:** 2019-12-21

**Authors:** Mayu Nonokawa, Ku Suzuki, Hideyuki Hayashi, Yuka Nishibata, Sakiko Masuda, Daigo Nakazawa, Satoshi Tanaka, Utano Tomaru, Akihiro Ishizu

**Affiliations:** 1 Department of Medical Laboratory Science, Faculty of Health Sciences, Hokkaido University, Kita-12, Nishi-5, Kita-ku, Sapporo, 0600812 Japan; 2 Department of Rheumatology, Endocrinology and Nephrology, Faculty of Medicine and Graduate School of Medicine, Hokkaido University, Sapporo, Japan; 3 Center for Cause of Death Investigation, Faculty of Medicine and Graduate School of Medicine, Hokkaido University, Sapporo, Japan; 4 Department of Pathology, Faculty of Medicine and Graduate School of Medicine, Hokkaido University, Sapporo, Japan

Dear Editor,

Myeloperoxidase-antineutrophil cytoplasmic antibody (MPO-ANCA) is a pathogenic autoantibody [1]. Wistar-Kyoto (WKY) rats immunized with human native MPO produce anti-human MPO antibody cross-reactive with rat MPO, resulting in the development of MPO-ANCA-associated vasculitis (MPO-AAV) [2]. MPO is a heterotetramer composed of two light chains (14 kDa) and two heavy chains (59 kDa) [3]. In this study, we examined if immunization of WKY rats with the recombinant light chain of human MPO could induce MPO-AAV.

WKY rats (4–5 weeks old) were immunized with the recombinant light chain of human MPO (1600 μg/kg; Cloud-Clone, Katy, TX, USA; group 1) or human native MPO (1600 μg/kg; RayBiotech, Peachtree Corners, GA, USA; group 2) on day 0. These rats were given an intraperitoneal injection of pertussis toxin (800 ng; Sigma-Aldrich, St. Louis, MO, USA) on days 0 and 2. A subgroup of group 1 was given an intraperitoneal injection of lipopolysaccharide (LPS; 100 μM/week; Sigma-Aldrich) through days 7 to 35. Urine samples were collected using a metabolic cage on day 40. All rats were euthanized on day 42.

Flow cytometry (FCM) using human neutrophils demonstrated the presence of ANCA in sera of group 2 but not group 1 (Fig. 1a). Correspondingly, sera of group 2 but not group 1 induced neutrophil extracellular traps (NETs) from tumor necrosis factor (TNF)-primed neutrophils (Fig. 1b). Immunoblot of neutrophil lysates demonstrated that antibody reactive with the MPO light chain (14 kDa) was produced in group 1, whereas antibodies reactive with the MPO heavy chain (59 kDa) and light chain (14 kDa) were produced in group 2 (Fig. 1c). The collective findings indicated that the anti-MPO light chain antibody produced in group 1 did not bind to native MPO. Renal tissue damage represented by hematuria and erythrocyte casts in renal tubules was evident in group 2 but not group 1 regardless of the disease boost by LPS (Fig. 1d, e). The degree of pulmonary hemorrhage that represents capillaritis in the lungs tended to be severe in group 2 compared to group 1 (Fig. 1f).

**Fig. 1:**
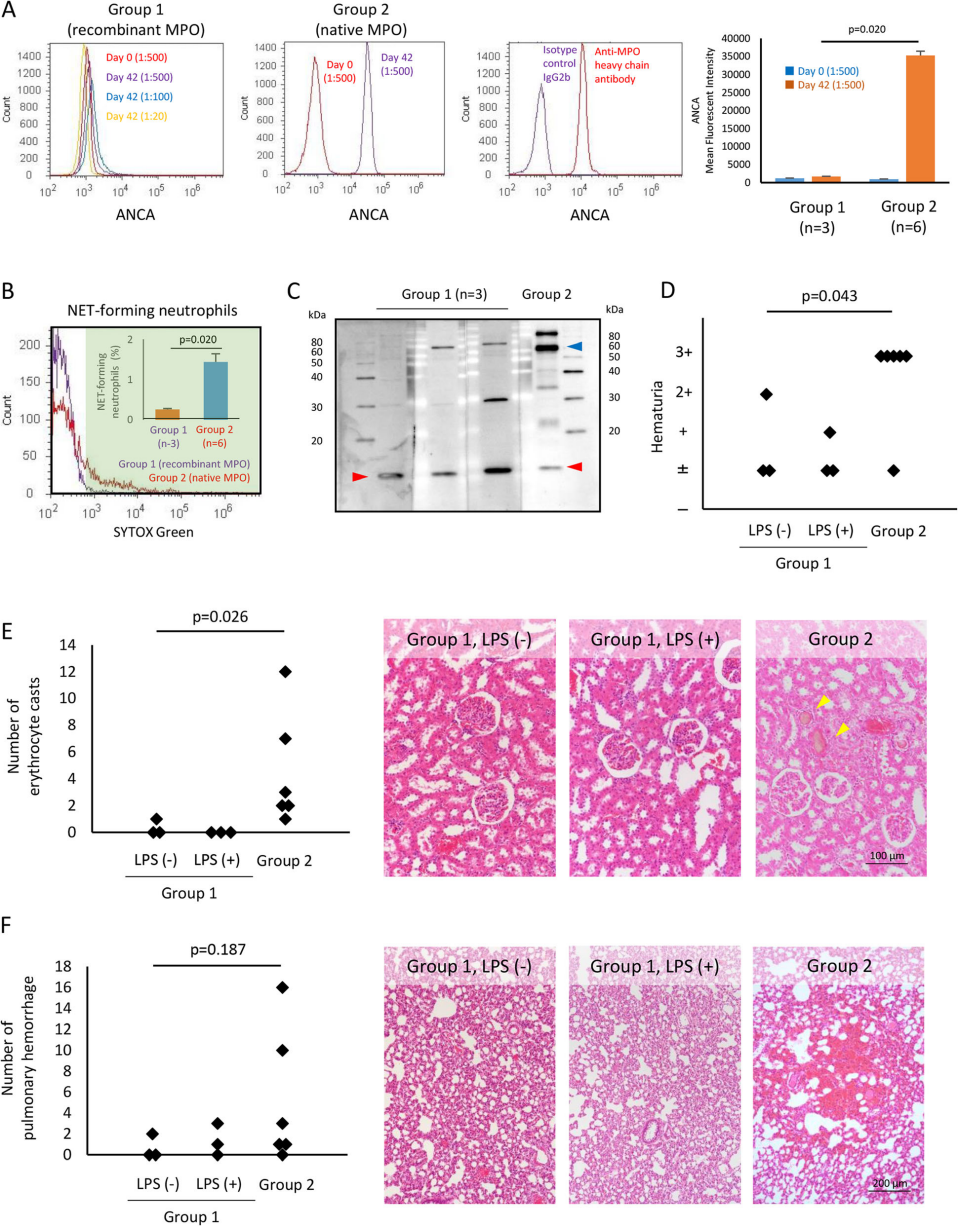
Development of MPO-AAV. **a** ANCA detected by FCM. Human peripheral blood neutrophils were fixed with 4% paraformaldehyde, and then the plasma membrane of neutrophils was penetrated using permeabilization wash buffer (BioLegend, San Diego, CA, USA). Cells (1 × 10^6^/ml) were allowed to react with 1:500 diluted rat sera for 30 min at room temperature (RT) followed by reaction with fluorescence-labeled secondary antibody. Concerning day 42 sera of group 1, the reactivity of 1:100 and 1:20 dilutions was also examined. To show the reactivity of anti-MPO heavy chain antibody to native MPO, a similar FCM was performed using the anti-MPO heavy chain monoclonal antibody (5 μg/ml; 4A4; Bio-Rad, Tokyo, Japan) as primary antibody and mouse IgG2b (5 μg/ml; BioLegend) as isotype control. **b** NET-forming neutrophils detected by FCM. Human peripheral blood neutrophils (1 × 10^6^/ml) were treated with 5 ng/ml TNF-α for 15 min at 37 °C and then exposed to 10% rat sera. After incubation for 3 h at 37 °C, cells were next made to react with a plasma membrane-impermeable DNA-binding dye, SYTOX Green (Life Technologies, Carlsbad, CA, USA). After filtering out the debris with a mesh, the percolated cells were subjected for FCM. Histograms highlighted in green represent NET-forming neutrophils. The percentage of NET-forming neutrophils induced by group 2 sera was significantly higher than that induced by group 1 sera. **c** ANCA detected by immunoblotting. Lysates of human neutrophils boiled under reducing condition were electrophoresed (5 × 10^5^ cells/lane) and then transferred to polyvinylidene difluoride membrane. After blocking the non-specific binding of antibodies, the membrane was incubated in diluted rat sera (day 42; group 1, 1:200 dilution; group 2, 1:1000 dilution) overnight at 4 °C. After rinsing with phosphate-buffered saline (PBS) with Tween 20 (PBS-T), the membrane was next incubated in the solution of horseradish peroxidase (HRP)-conjugated secondary antibody for 1 h at RT. After rinsing with PBS-T, the HRP activity on the membrane was detected by chemiluminescence using ImageQuant LAS 4000 (GE Healthcare, Little Chalfont, UK). Blue arrowhead, MPO heavy chain (59 kDa); red arrowheads, MPO light chain (14 kDa). **d** Degree of hematuria assessed at urine sampling immediately by a dipstick (Siemens Healthineers, Erlangen, Germany). **e** Degree of renal tissue damage. Erythrocyte casts (yellow arrowheads) were counted in the maximum longitudinal section of the kidney. **f** Degree of pulmonary hemorrhage. The foci of pulmonary hemorrhage were counted in the maximum longitudinal section of the lung. Mann-Whitney *U* test was applied for statistical analyses between two non-parametric groups

The majority of MPO-AAV patients produced MPO-ANCA that recognizes an epitope in the heavy chain of MPO, whereas a few number of patients produced MPO-ANCA against an epitope in the light chain of MPO [4, 5]. The collective findings suggested that the recombinant light chain of human MPO has a low potential to induce MPO-AAV in rats compared to native human MPO.

## Data Availability

The data sheets used and/or analyzed during the current study are available from the corresponding author on reasonable request.
